# Biotransformation of 2-keto-4-hydroxybutyrate via aldol condensation using an efficient and thermostable carboligase from *Deinococcus radiodurans*

**DOI:** 10.1186/s40643-024-00727-x

**Published:** 2024-01-16

**Authors:** Yeon-Ju Jeong, Min-Ju Seo, Bong Hyun Sung, Jeong-Sun Kim, Soo-Jin Yeom

**Affiliations:** 1 School of Biological Sciences and Biotechnology, Graduate School, Chonnam National University, Gwangju, Republic of Korea; 2 School of Biological Sciences and Technology, Chonnam National University, Gwangju, Republic of Korea; 3 Institute of Synthetic Biology for Carbon Neutralization, Chonnam National University, Gwangju, 61186 Republic of Korea; 4 Synthetic Biology Research Center, Korea Research Institute of Bioscience and Biotechnology, Daejeon, 34141 Republic of Korea; 5 Department of Chemistry, Chonnam National University, Gwangju, 61186 Republic of Korea

**Keywords:** Formaldehyde, Pyruvate, Pyruvate aldolase, 2-keto-4-hydroxybutyrate, *Deinococcus radiodurans*

## Abstract

The bioconversion of 4-hydroxy-2-keto acid derivatives via aldol condensation of formaldehyde and pyruvate has received substantial attention as potential source of chemicals for production of amino acids, hydroxy carboxylic acids, and chiral aldehydes. We developed an environmentally friendly biocatalyst consisting of a novel thermostable class II pyruvate aldolase from *Deinococcus radiodurans* with maltose-binding protein (MBP-DrADL), which has specific activity of 46.3 µmol min^–1^ mg^–1^. Surprisingly, MBP-DrADL maintained over 60% of enzyme activity for 4 days at 50 to 65 °C, we used MBP-DrADL as the best candidate enzyme to produce 2-keto-4-hydroxybutyrate (2-KHB) from formaldehyde and pyruvate via aldol condensation. The optimum reaction conditions for 2-KHB production were 50 °C, pH 8.0, 5 mM Mg^2+^, 100 mM formaldehyde, and 200 mM pyruvate. Under these optimized conditions, MBP-DrADL produced 76.5 mM (8.94 g L^–1^) 2-KHB over 60 min with a volumetric productivity of 8.94 g L^–1^ h^–1^ and a specific productivity of 357.6 mg mg-enzyme^–1^ h^–1^. Furthermore, 2-KHB production was improved by continuous addition of substrates, which produced approximately 124.8 mM (14.6 g L^–1^) of 2-KHB over 60 min with a volumetric productivity and specific productivity of 14.6 g L^–1^ h^–1^ and 583.4 mg mg-enzyme^–1^ h^–1^, respectively. MBP-DrADL showed the highest specific productivity for 2-KHB production yet reported. Our study provides a highly efficient biocatalyst for the synthesis of 2-KHB and lays the foundation for large-scale production and application of high-value compounds from formaldehyde.

**Graphical Abstract:**

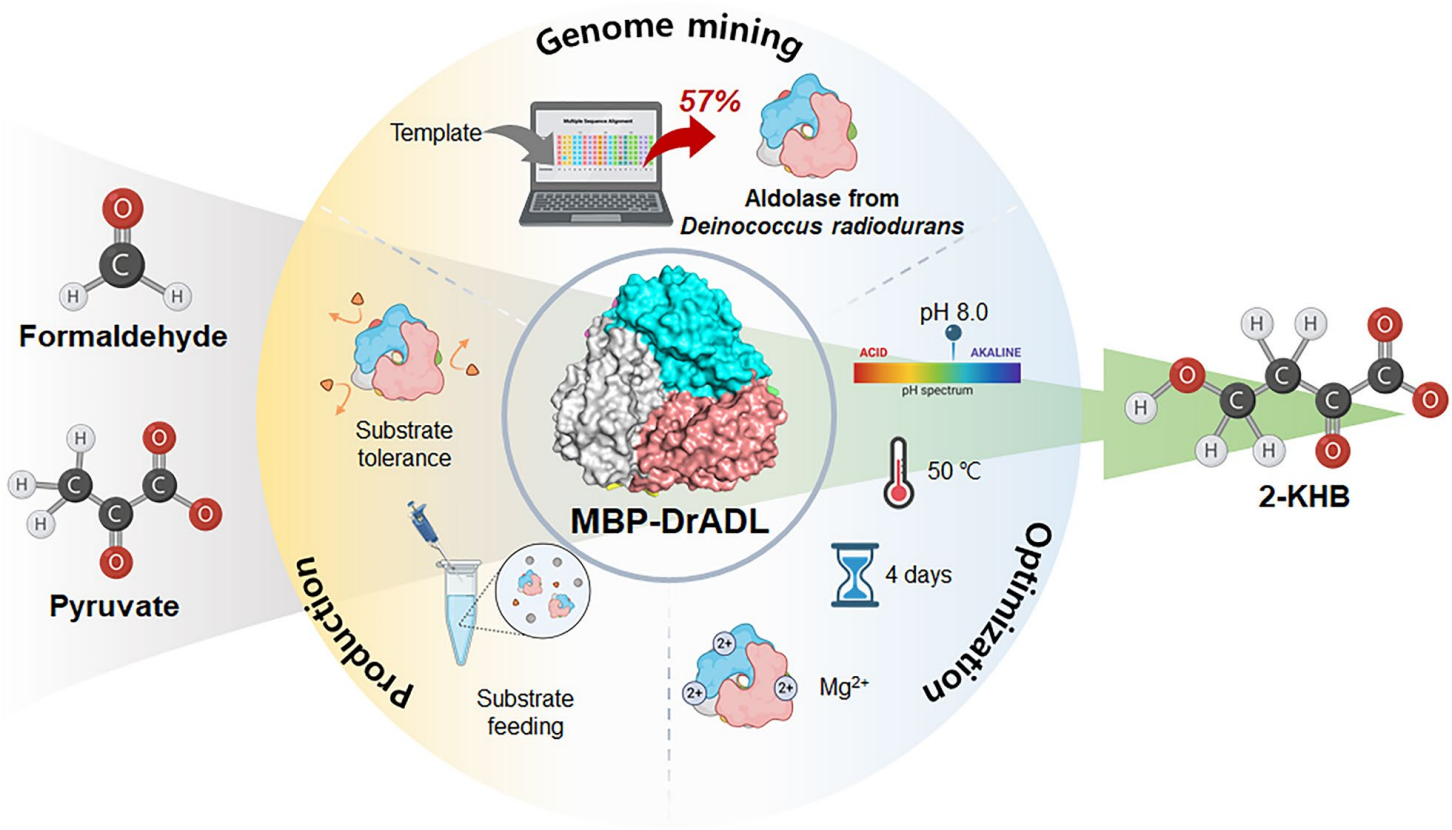

**Supplementary Information:**

The online version contains supplementary material available at 10.1186/s40643-024-00727-x.

## Introduction

The high-value utilization of one-carbon compounds has been gaining attention recently as a low-cost, abundant feedstock option. One of the biggest challenges in realizing the one-carbon (C1) chemistry is the synthesis of complex carbon compounds from C1 building blocks. Formaldehyde is an emerging C1 resource since it can be easily derived from carbon monoxide, carbon dioxide, formic acid, methane, and methanol by biological or chemical means (Desmons et al. 2019; Jo et al. 2022). Formaldehyde-converting enzymes such as formolase, benzaldehyde lyase, and pyruvate aldolase are the key biocatalysts in the synthesis of value-added chemicals from C1 feedstocks (Güner et al. 2021; Hernandez et al. 2017; Li et al. 2023; Siegel et al. 2015). Despite considerable research on this subject, the progress achieved so far has not translated into highly efficient catalyst systems or processes that enable large-scale industrial application. It is very, therefore, crucial to discover new high-performing enzymes that can be applied to the industrialized production of resources such as biomass, industrial by-products, and fossil fuels.

Aldol condensation by aldol addition using carboligating enzymes is a powerful strategy that enables carbon–carbon bond formation in a sustainable and environmentally friendly fashion. Direct aldol additions mediated by aldolases are finding increasing application in chemical research and in the production of asymmetric compounds due to their high selectivity and catalytic efficiency (Gastaldi et al. 2022; Gong et al. 2021; Hartley et al. 2017; Ju et al. 2022; Liu et al. 2023; Meng et al. 2021; Sugiyama et al. 2007). One way to convert formaldehyde is through aldol condensation, which can be achieved by pyruvate aldolase. Pyruvate aldolase reversibly mediates the C–C bond formation between pyruvate and aldehydes to produce 4-hydroxy-2-keto acids, which are relatively little exploited in organic synthesis. The aldol addition of pyruvate to formaldehyde is especially relevant because 2-KHB, one of the resulting aldol adducts, is an intermediate of l-homoserine, 3-hydroxypropionaldehyde, and 1,3-propanediol derivatives, which are important starting materials in the manufacture of biocompatible plastic and polytrimethylene terephthalate (Bouzon et al. 2017; Cesnik et al. 2020; Chen et al. 2015; Frazao et al. 2019; Katulic et al. 2021; Wang et al. 2019; Xu et al. 2020; Zhang et al. 2019; Zhong et al. 2019).

In recent years, several class II pyruvate aldolases have been reported and engineered to develop powerful biocatalysts that lead to the organic synthesis of enantiomerically pure formulations via aldol condensation of aldehyde with carbonyl donors, which have potential to catalyze the stereochemical formation of C–C bonds (Baker and Seah 2012; Dekker et al. 1971; Royer et al. 2010; Wang et al. 2010a; Williams et al. 2006). Specifically, these enzymes have primarily been studied utilizing aldehydes with straight or branched chain consisting of two to five carbons including acetaldehyde, glycolaldehyde, propionaldehyde, and glyceraldehyde (Baker and Seah 2012; Laurent et al. 2019; Wang et al. 2010a).

Only a few pyruvate aldolases for conversion of 2-KHB lacking enantiomeric properties have been reported such as 2-keto-3-deoxy-l-rhamnonate aldolase from *Escherichia coli* with maltose-binding protein (MBP-EcYfaU), 2-keto-4-hydroxybutyrate aldolase from *E. coli* K-12 (EcKHB), and 2-keto-4-hydroxyglutarate aldolase from *Bos taurus* (BtKHG), *Homo sapiens* (HsKHG), and *Rattus norvegicus* (RnKHG) (Wang et al. 2019). Among them, MBP-EcYfaU is unique in having been rationally engineered for formaldehyde tolerate, thermostability, and stereospecificity, and used for the production of amino acids via a multi-enzyme cascade reaction (Bosch et al. 2021; Hernandez et al. 2017, 2018). MBP-EcYfaU was also applied to generate formaldehyde-assimilating synthetic bacteria (He et al. 2020; Wang et al. 2019). However, biotransformation of formaldehyde remains challenging because of the substance’s toxicity and symmetrical reactivity (Desmons et al. 2019; Teng et al. 2001). Formaldehyde leads to enzyme inactivation through its interaction with nucleophilic residues (Feldman 1973; Hansen et al. 1974). Recent, studies have reported that elevated aldolase stability, achieved through approaches like immobilization or improved thermal stability, results in increased resistance to aldehydes (Dick et al. 2016; Fei et al. 2014; Nara et al. 2011). The heightened thermal stability of the enzyme can be attributed to its structural stability, which also contributes to its aldehyde resistance.

**Scheme 1:**
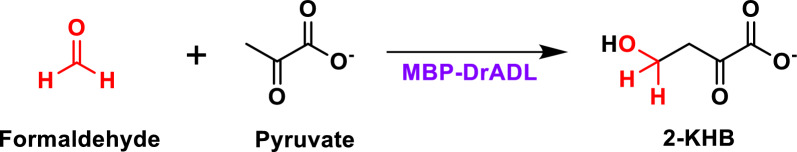
Biosynthesis of 2-KHB from formaldehyde and pyruvate using MBP-DrADL

Recently, we reported the use of pyruvate aldolase from *Pseudomonas aeruginosa* with maltose-binding protein (MBP-PaADL) for 2-KHB production from formaldehyde and pyruvate (Jeong et al. 2023). In the previous study, MBP-PaADL produced the highest 2-KHB yield reported so far. The determination of an efficient new pyruvate aldolase is required to further raise this bar and constitutes an important prerequisite for the utilization and assimilation of C1 compounds. We here describe a new thermostable pyruvate aldolase from *Deinococcus radiodurans* with maltose binding protein (MBP-DrADL), which showed the highest specific productivity for 2-KHB production reported to date. The enzymatic properties of recombined MBP-DrADL were systematically characterized, including optimum temperature, pH, and metal ion. We also confirmed the thermostability of the enzyme. In addition, continuous low-concentration formaldehyde feeding was demonstrated to increase 2-KHB production without increasing formaldehyde toxicity. Under these optimized reaction conditions, an increase in the 2-KHB production from formaldehyde and pyruvate was achieved (Scheme 1).

## Materials and methods

### Gene cloning

The gene encoding DrADL (GenBank accession no. AAF12475.1, NCBI) was commercially synthesized by Cosmo Genetech (Seoul, Republic of Korea) (Additional file 1: Table S1). For the pLIC.B4-DrADL, DrADL gene was amplified using polymerase chain reaction (PCR). The sequences of the primers used for gene cloning were based on the DNA sequence of DrADL. Forward (5ʹ-GGG CGG CGG TGG TGG CGG CAT GCC GCA GCC GAT G-3ʹ), reverse (5ʹ-CAG TTC TTC TCC TTT GCG CCC CTA GTA AAC AGA AC-3ʹ); forward (5ʹ-GTT CTG TTT ACT AGG GGC GCA AAG GAG AAG AAC TG-3ʹ), and reverse (5ʹ-CAT CGG CTG CGG CAT GCC GCC ACC ACC GCC GCC C-3ʹ) primers were designed to amplify DrADL DNA fragments and expression vector (pLIC.B4 including His_6_, maltose-binding protein (MBP), and tobacco etch virus protease cleavage site at the N-terminus) (Aslanidis and de Jong 1990), respectively, and were synthesized by Macrogen facility (Daejeon, Republic of Korea). The amplified fragments were ligated using Gibson assembly master mix (New England Biolabs, Ipswich, MA, USA) (Gibson et al. 2009). The ligated fragments were transformed into *E. coli* DH5α and plated on Luria–Bertani (LB) agar containing 50 μg mL^–1^ ampicillin. The selected colony was isolated using a plasmid purification kit (Qiagen) and sequenced at the Macrogen facility (Daejeon, Republic of Korea). The sequenced plasmid was transformed into *E. coli* C2566 for protein expression and purification.

### Expression and purification

After transformation of the constructed recombinant plasmid into *E. coli* C2566, which were grown in LB media containing 50 μg mL^–1^ ampicillin at 200 rpm at 37 °C. When OD_600_ reached 0.6, the expression of protein was induced by adding 50 μM isopropyl-β-d-thiogalactopyranoside (final concentration), then continuously incubated for 16 h with shaking at 150 rpm at 20 °C. The culture was harvested by centrifugation at 3800 rpm at 4 °C for 20 min. The harvested cells were resuspended in an ice-cold lysis buffer containing 20 mM Tris–HCl (pH 7.9) buffer with 500 mM NaCl and disrupted by sonication. The supernatant was collected by centrifugation at 13,000 rpm for 20 min at 4 °C and loaded onto a Ni-charged immobilized metal ion affinity chromatography column (BioWorks, Uppsala, Sweden). The bound proteins were washed twice with lysis buffer containing 10 mM and 20 mM imidazole and eluted by 250 mM of imidazole in the lysis buffer. The eluted proteins were dialyzed against 50 mM *N*-(2-hydroxyethyl)piperazine-*Nʹ*-(3-propanesulfonic acid) (EPPS, pH 8.0) containing 1 mM Mg^2+^. The protein concentration was determined by the Bradford assay using bovine serum albumin as standard (Bradford 1976). Sodium dodecyl sulphate polyacrylamide gel electrophoresis (SDS-PAGE) was performed according to the method described by Laemmli (Laemmli 1970).

### Biochemical properties of MBP-DrADL

To evaluate the effect of pH on enzyme activity, pH values were varied from 6.5 to 10.0 using 50 mM piperazine-*N*,*N*ʹ-bis(2-ethanesulfonic acid) (PIPES, pH 6.5–7.5), 50 mM EPPS (pH 7.5–8.5), and 50 mM *N*-cyclohexyl-2-aminoethanesulfonic acid (CHES, pH 8.5–10.0) with 1 mM Mg^2+^, 5 mM formaldehyde, and 5 mM pyruvate for 10 min at 50 °C. The effect of temperature on enzyme activity was monitored at different temperatures (20–55 °C in 5 °C increments) in 50 mM EPPS (pH 8.0) buffer containing 0.002 mg mL^–1^ enzyme, 1 mM Mg^2+^, 5 mM formaldehyde, and 5 mM pyruvate for 10 min. For thermostability testing, the enzyme was incubated at 50, 55, 60, and 65 °C for a maximum of 7 days without substrate, and then residual activity of MBP-DrADL was measured. To investigate the effect of metal ions on enzyme activity, an enzyme assay was carried out after treatment with 1 mM ethylenediaminetetraacetic acid (EDTA) at 4 °C for 1 h or after the addition of 1 mM of each metal ion (Mg^2+^, Mn^2+^, Co^2+^, Zn^2+^, Ni^2+^, Cu^2+^, or Ca^2+^). The reactions were performed in 50 mM EPPS buffer (pH 8.0) containing each metal ion with 5 mM formaldehyde and 5 mM pyruvate for 10 min at 50 °C. To determine the effect of Mg^2+^ concentration, the Mg^2+^ concentration was varied from 0.5 to 10 mM in 50 mM EPPS (pH 8.0) containing 0.002 mg mL^–1^ enzyme, 5 mM formaldehyde, and 5 mM pyruvate for 10 min.

### Determination of aldol condensation activity

Specific activity of MBP-DrADL was investigated at 50 °C and pH 8.0 with 50 mM EPPS containing 0.002 mg mL^–1^ enzyme, 5 mM Mg^2+^, 5 mM formaldehyde, and 5 mM pyruvate for 10 min. One unit (U) of aldolase was defined as the amount of enzyme required to produce 1 μmol of 2-KHB per min. The specific activity of the enzyme was defined as the produced amount of product per unit reaction time per enzyme amount. Formaldehyde, pyruvate, and 2-KHB were quantitatively determined by high performance liquid chromatography (HPLC) as described in our previous study (Jeong et al. 2023).

### Kinetic parameter

Apparent kinetic parameters of MBP-DrADL, for formaldehyde (1–30 mM) and pyruvate (1–30 mM) were determined in a steady-state assumption of 10 min reactions in 50 mM EPPS buffer (pH 8.0) containing each 0.002 mg mL^−1^ enzyme and 5 mM Mg^2+^ at 50 °C. For the kinetics, one of the two substrates was fixed with 5 mM and the other substrate was varied. The *k*_cat_ and *K*_m_ values were determined by nonlinear regression with the Michaelis–Menten equation using the GraphPad Prism 6 software (GraphPad Software, San Diego, CA, USA).

### Optimization of reaction conditions for 2-KHB production

Unless otherwise stated, the reaction was performed in 50 mM EPPS buffer (pH 8.0) with 5 mM Mg^2+^ at 50 °C for 2 h. To determine the optimal concentration of MBP-DrADL, 0.005 to 0.1 mg mL^–1^ enzyme was incubated with 100 mM formaldehyde and 100 mM pyruvate. The optimal substrate concentration was determined with 0.025 mg mL^–1^ MBP-DrADL by 10–300 mM formaldehyde or pyruvate by fixing another substrate at 100 mM. Time-course reactions with single or continuous batches were made with 0.025 mg mL^–1^ of MBP-DrADL, 100 mM formaldehyde, and 200 mM pyruvate. The substrate feeding was conducted by adding 50 mM formaldehyde and 50 mM pyruvate simultaneously ay intervals of 10 min under the conditions described above.

### LC–MS analysis for by-product identification

Identification of by-product by aldolase with formaldehyde and pyruvate as substrate was performed by LC–MS. The instrumentation consisted of an Agilent 1290 Infinity UPLC system and a Agilent 6550 iFunnel Q-TOF LC/MS (G6550A) mass spectrometer. A Waters Acquity C18 column (2.1 mm × 50 mm, 1.8 μm) at 30 °C was used for separation with gradient elution (phase A, 0.1% formic acid in water and phase B, 0.1% formic acid in acetonitrile) using the following linear gradient: 0–30 min (5–80% B in A), 30–35 min (80% B), 35–37 min (80–5% B), and the column was then equilibrated with 5% of B for 5 min at a flow rate of 0.3 mL/min. Samples of 1 μL were injected into the column using an autosampler. The HPLC system was interfaced to the MS system, a Dual AJS ESI equipped with an ESI source. The ion spray voltage was 4.0 kV for positive ions and 3.5 kV for negative ions. Mass ranges from 80 to 1700 m/z in ESI mode were obtained.

## Results and discussion

### Identification of MBP-DrADL

**Fig. 1:**
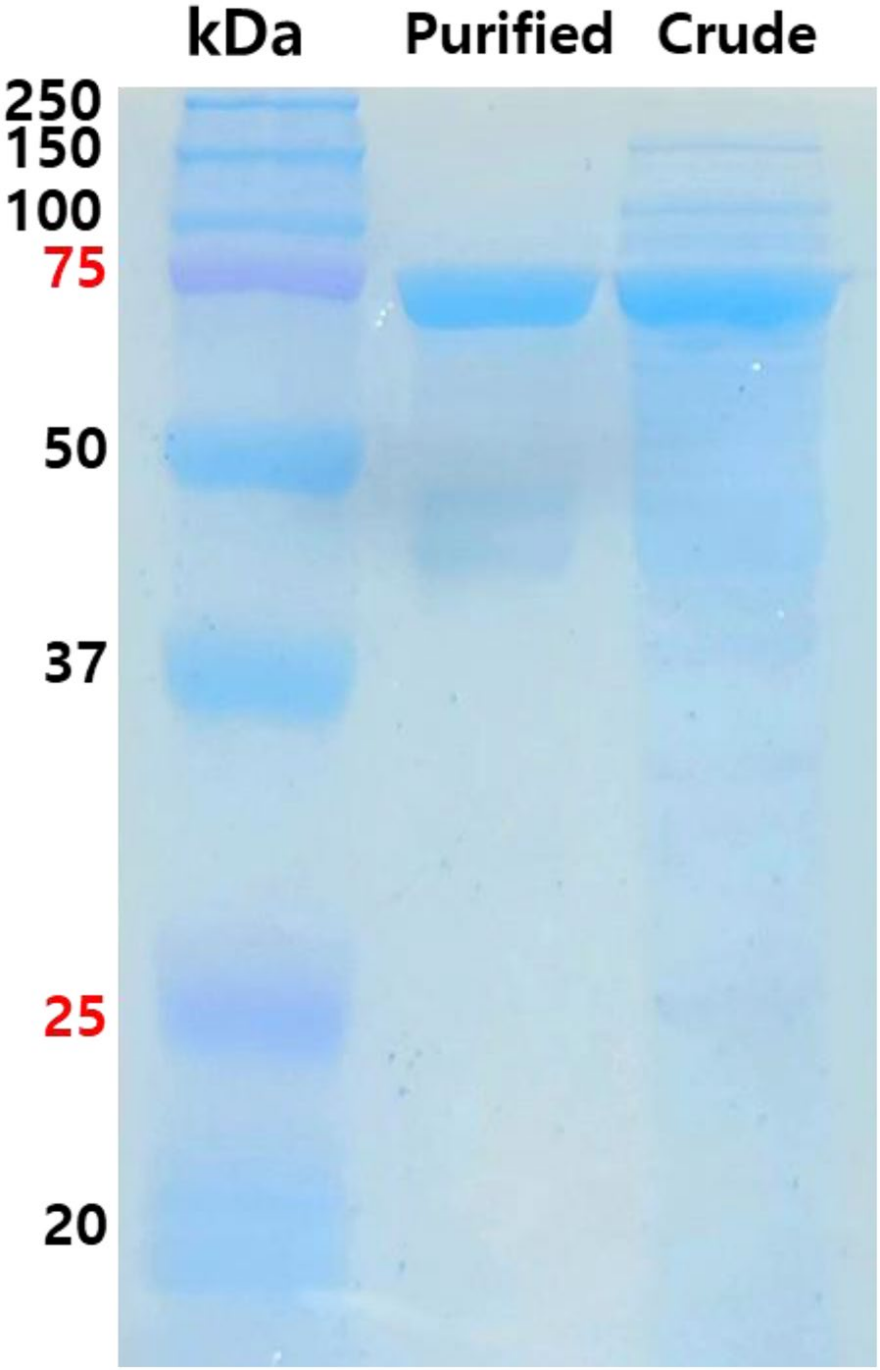
SDS-PAGE analysis of MBP-DrADL. Protein markers (250, 150, 100, 75, 37, and 25 kDa), crude extract, and purified enzyme were loaded

In the present study, genome mining was used to discover new pyruvate aldolase with high activities and substrate affinities via bioinformatics analysis based on the NCBI database. Using EcYfaU as the templates, alignment of amino acid sequences was conducted with candidate genes from a wide range of microbes. We ultimately selected DrADL, which showed 57.0% and 67.4% identity with EcYfaU and PaADL, respectively. The molecular weight of the recombinant MBP-DrADL as estimated by SDS-PAGE was approximately estimated 73.0 kDa (Fig. 1).

**Fig. 2:**
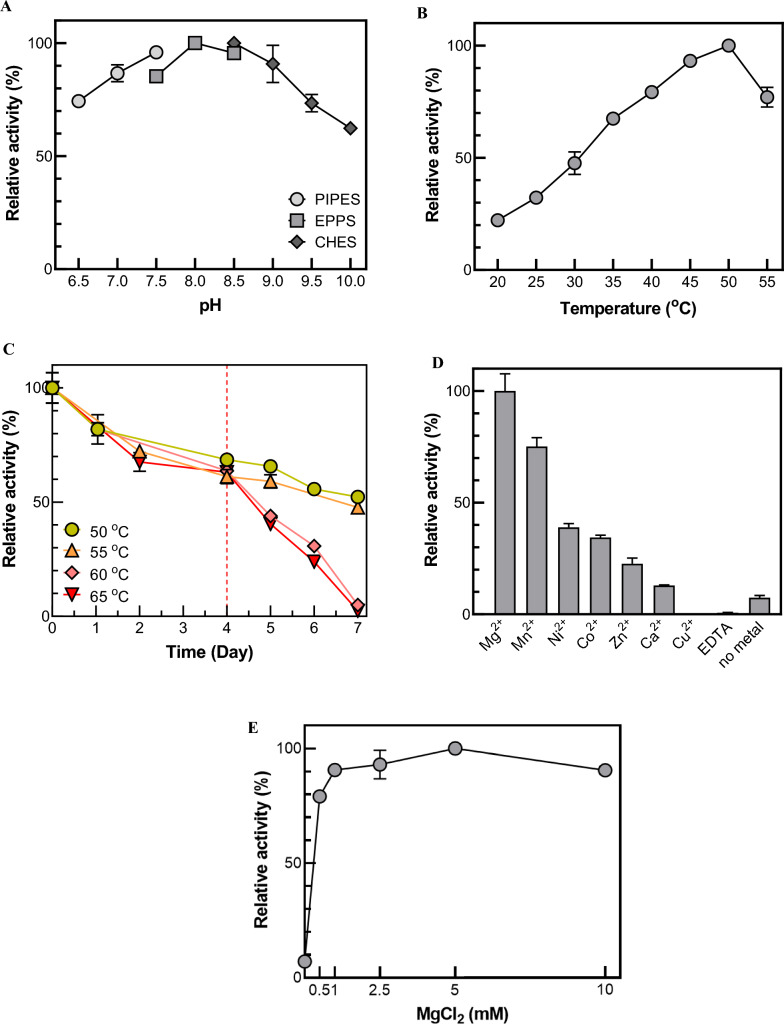
Effects of pH, temperature, and metal ions on MBP-DrADL activity. **A** Effect of pH. The reactions were performed using three different types of buffers: PIPES (pH 6.5–7.5), EPPS (pH 7.5–8.5), and CHES (pH 8.6–10.0). **B** Effect of temperature. **C** Thermostability of MBP-DrADL. The reactions were performed with 0.002 mg mL^−1^ enzyme, 5 mM formaldehyde, and 5 mM pyruvate in the presence of 1 mM Mg^2+^ for 10 min. **D** Effect of different metal ions. **E** Effect of Mg^2+^ concentration. The reactions were conducted in 50 mM EPPS (pH 8.0) buffer containing 0.002 mg mL^−1^ enzyme, 5 mM formaldehyde, and 5 mM pyruvate at 50 °C for 10 min

To acquire the values for optimum reaction conditions, enzymatic activities of MBP-DrADL at various pH values and temperatures were measured. The enzyme activity in converting formaldehyde and pyruvate to 2-KHB was measured by analyzing a reaction mixture using HPLC. MBP-DrADL showed maximum activity at pH 8.0 in EPPS buffer (Fig. 2A) and at 50 °C (Fig. 2B). Notably, MBP-DrADL maintained a high enzyme activity of > 70% under broad pH condition (pH 6.5–9.5). The enzyme activity of MBP-EcYfaU was tested at 25 °C (Hernandez et al. 2017) or 30 °C in pH 7.0 (Wang et al. 2019), and the optimum conditions of MBP-PaADL were 45 °C and pH 9.0 (Jeong et al. 2023). Moreover, the enzymatic activities of other pyruvate aldolases including EcKHB, BtKHG, HsKHG, and RnKHG were measured at 30 ℃ and at pH 7.0 (Hernandez et al. 2017; Wang et al. 2019) (Table 1). These results suggest that there are significant differences in the catalytic properties of MBP-DrADL from different pyruvate aldolases.

**Table 1:** Biochemical properties of the characterized pyruvate aldolases

Enzyme	Temperature (°C)	pH	Specific activity (μmol mg^−1^ min^−1^)	Formaldehyde			Pyruvate			References
				*k*_cat_ (min^−1^)	*K*_m_ (mM)	*k*_cat_/*K*_m_ (min^−1^ mM^−1^)	*k*_cat_ (min^−1^)	*K*_m_ (mM)	*k*_cat_/*K*_m_ (min^−1^ mM^−1^)	
MBP-EcYfaU	25^a^	7.0^a^	10	NR	NR	NR	NR	NR	NR	Hernandez et al. (2017)
MBP-EcYfaU	25^a^	7.0^a^	60 ± 1^b^	113	24	4.71	113	209	0.54	Bosch et al. (2021)
EcKHB	30^a^	7.0^a^	1.62 ± 0.11^c^	NR	NR	NR	NR	NR	NR	Wang et al. (2019)
BtKHG	30^a^	7.0^a^	0.11 ± 0.01^c^	NR	NR	NR	NR	NR	NR	Wang et al. (2019)
HsKHG	30^a^	7.0^a^	0.10 ± 0.01^c^	NR	NR	NR	NR	NR	NR	Wang et al. (2019)
RnKHG	30^a^	7.0^a^	0.08 ± 0.01^c^	NR	NR	NR	NR	NR	NR	Wang et al. (2019)
MBP-PaADL^WT^	45	9.0	5.87 ± 0.05	271	45	10	255	37	6.8	Jeong et al. (2023)
MBP-PaADL^V121A/L241A^	45	9.0	7.54 ± 0.05	398	65	11	453	52	8.8	Jeong et al. (2023)
MBP-DrADL	50	8.0	46.3 ± 0.36	11,845	8.79	1347	7670	4.06	1889	This study

NR: not reported

^a^The enzyme activities were assayed each condition

^b^Specific activity of MBP-EcYfaU was determined with retro-aldol consultation activity

^c^Enzyme activities of these enzymes were measured using resting cells expressing each enzyme (unit = μmol min^−1^ OD^−1^ mg^−1^)

MBP-DrADL exhibited the highest activity at slightly higher temperature than other pyruvate aldolases, confirming its thermostability for 2-KHB production. For the thermostability test, the enzyme was pre-incubated at various temperatures (50, 55, 60, and 65 °C), and aliquots were withdrawn for each interval to test the residual activities. The activity of MBP-DrADL was retained above 60% after incubation for four days independent of temperature; after that period, substantial differences in thermostability developed (Fig. 2C). MBP-EcYfaU, which was engineered to increase thermostability, has a half-life of 11 h. MBP-DrADL displayed 8.7-fold higher thermo-stability than MBP-EcYfaU, indicating that MBP-DrADL might be considered for continuous 2-KHB production in future applications.

Class II aldolases are divalent metal ion-dependent metalloenzymes (Hixon et al. 1996; Rea et al. 2008). To determine the effect of metal ions on the catalytic activity of the enzyme, various kinds of metal ions or EDTA (1 mM) were supplemented into the enzyme solutions for 24 h of incubation before analysis of enzymatic activities. Among the tested ions, Mg^2+^ yielded the highest MBP-DrADL activity (Fig. 2D) at an optimal concentration of 5 mM (Fig. 2E). The catalytic activity of the other aldolases was also ion dependent, with Co^2+^ acting on MBP-EcYfaU (Hernandez et al. 2017) and Mg^2+^ on MBP-PaADL (Jeong et al. 2023), indicating a diversity of metal dependencies even among aldolases that catalyze the same chemical reactions.

### Aldol condensation activities of aldolases

The next steps were to confirm the catalytic activities to select the most promising enzymes. Therefore, the specific activity of MBP-DrADL was compared to wild-type of MBP-PaADL (MBP-PaADL^WT^) and a variant of MBP-PaADL (MBP-PaADL^V121A/L241A^) under optimal conditions for each enzyme (Jeong et al. 2023). The reaction conditions for MBP-PaADL were at pH 9.0 and 45 ℃ in presence of 1 mM Mg^2+^ (2017; Jeong et al. 2023). In our previous study, the specific activities of MBP-PaADL^WT^ and MBP-PaADL^V121A/L241A^ were reported as 5.87 and 7.54 nmol min^–1^ mg^–1^, respectively; however, this was in error and these values should be corrected to µmol min^–1^ mg^–1^, respectively (Jeong et al. 2023). In previous study, the specific activity of MBP-EcYfaU was 10 µmol min^–1^ mg^–1^ (Hernandez et al. 2017). To compare aldol condensation activities MBP-DrADL with other pyruvate aldolase, we tested the activity assay using MBP-DrADL at pH 8.0 and 50 ℃ in the presence of 5 mM Mg^2+^. MBP-DrADL showed 4.63-fold, 7.9-fold, and 6.1-fold higher aldol specific activity than that of MBP-EcYfaU, MBP-PaADL^WT^, and MBP-PaADL^V121A/L241A^, respectively (Table 1). This demonstrates that MBP-DrADL was most efficient in catalyzing the condensation of formaldehyde and pyruvate which is a general property of known class II pyruvate aldolases. We, therefore, used MBP-DrADL in the following experiments, as having the highest specific activity for 2-KHB production from formaldehyde and pyruvate.

### Kinetic parameters of MBP-DrADL

The kinetic parameters of MBP-DrADL were determined toward formaldehyde and pyruvate (Table 1). In previous study, MBP-PaADL^V121A/L241A^ showed the highest *k*_cat_ values for formaldehyde (398 min^−1^) and pyruvate (453 min^−1^) among the reported pyruvate aldolases (Table 1). The *k*_cat_ values of MBP-DrADL for formaldehyde and pyruvate were 11,845 and 7670 min^−1^, respectively, which were 29.8- and 16.9-fold higher than *k*_cat_ values of MBP-PaADL^V121A/L241A^, respectively. Moreover, the *K*_m_ values of MBP-DrADL toward formaldehyde and pyruvate were 8.79 mM and 4.06 mM, respectively, which were 7.4- and 12.8-fold lower than MBP-PaADL^V121A/L241A^, respectively (Jeong et al. 2023) (Table 1). MBP-DrADL showed the highest catalytic efficiencies for formaldehyde (1347 min^−1^ mM^−1^) and pyruvate (1889 min^−1^ mM^−1^), respectively, which were 122.5- and 214.7-fold higher than MBP-PaADL^V121A/L241A^, respectively; and 286- and 3498-fold higher than MBP-EcYfaU, respectively (Bosch et al. 2021; Jeong et al. 2023) (Table 1).

### Effects of enzyme and substrate concentrations

**Fig. 3:**
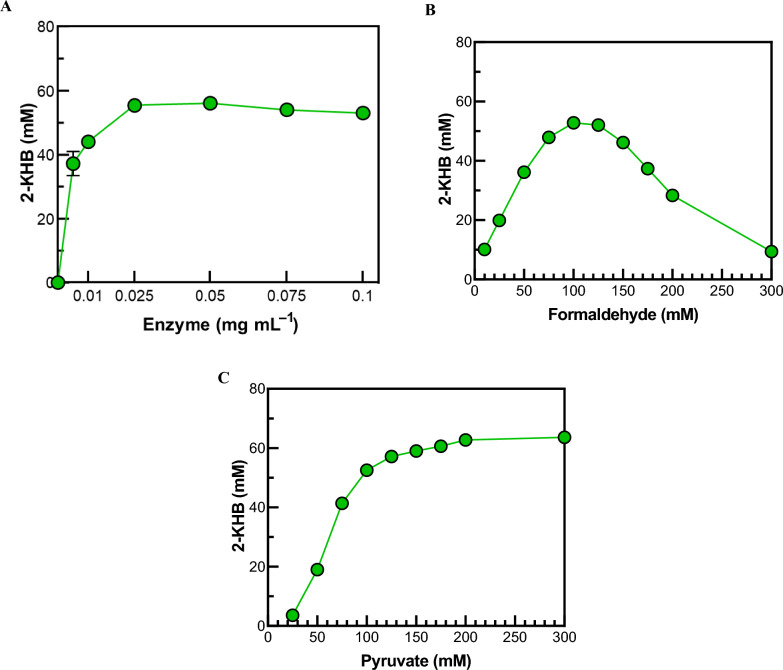
Effects of enzyme and substrate concentrations on 2-KHB production from formaldehyde and pyruvate. **A** Effect of enzyme concentration. **B** Effect of formaldehyde concentration. **C** Effect of pyruvate concentration. The reactions were performed different concentrations of enzyme substrates in 50 mM EPPS (pH 8.0) buffer with 5 mM Mg^2+^ at 50 °C for 10 min

To investigate the optimal enzyme concentration for the 2-KHB production, 0.005–0.1 mg mL^−1^ MBP-DrADL was incubated with 100 mM formaldehyde and 100 mM pyruvate in the presence of 5 mM Mg^2+^. When the enzyme concentration was below 0.025 mg mL^−1^, the amount of 2-KHB increased with enzyme concentration. However, above 0.025 mg mL^−1^ enzyme, 2-KHB concentration reached a saturation state, and plateaued at approximately 55.5 mM (Fig. 3A). Next, we determined the optimal substrate concentrations for the reaction. When 0–300 mM formaldehyde or pyruvate were tested with 0.025 mg mL^−1^ enzyme, the maximal production of 2-KHB occurred at a concentration of 100 mM formaldehyde and 200 mM pyruvate (Fig. 3B and 3C). At pyruvate concentrations above 200 mM, the amount of 2-KHB reached a plateau, whereas it decreased with an increase in formaldehyde concentration at concentrations above 100 mM. It can thus be concluded that 2-KHB production was inhibited by formaldehyde concentration. This phenomenon is consistent with MBP-PaADL, while MBP-EcYfaU exhibited an inhibitory effect on 2-KHB production at formaldehyde and pyruvate concentrations of 80 mM and 40 mM, respectively (Jeong et al. 2023).The thermal stability of aldolases, illustrated by their optimal reaction temperatures, appears to be correlated with their resilience to substrate inhibition.

### Biotransformation of 2-KHB using MBP-DrADL

**Fig. 4:**
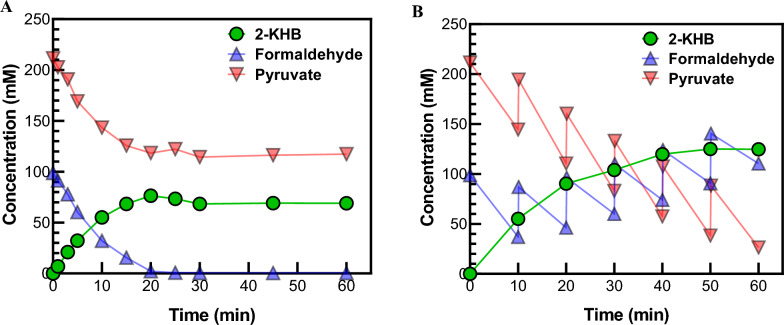
High production of 2-KHB from formaldehyde and pyruvate using MBP-DrADL. **A** Time-course reactions without batch-feeding substrates. The reactions were performed in 50 mM EPPS (pH 8.0) buffer containing 0.025 mg mL^−1^ enzyme, 100 mM formaldehyde, 200 mM pyruvate, and 5 mM Mg^2+^ at 50 °C for 1 h. **B** Time-course reactions while batch-feeding substrates. The reactions were performed under the same condition as **A**, but 50 mM formaldehyde and 50 mM pyruvate were added every 10 min up to 60 min

The 2-KHB production from formaldehyde and pyruvate using MBP-DrADL was performed at pH 8.0, 50 °C, 0.025 mg mL^−1^ enzyme, 100 mM formaldehyde, and 200 mM pyruvate in the presence of 5 mM Mg^2+^. Under optimized reaction conditions, the time-course reactions were conducted using MBP-DrADL, which produced 76.5 mM (8.94 g L^−1^) of 2-KHB from 100 mM formaldehyde and 200 mM pyruvate for 60 min, corresponding to a volumetric productivity of 8.94 g L^−1^ h^−1^ and a specific productivity of 357.6 mg mg-enzyme^−1^ h^−1^ (Fig. 4A). However, after 20 min, the 2-KHB production did not increase because of exhausted formaldehyde. Also, we observed that a high concentration of formaldehyde could inhibit the reaction and reduce the yield of 2-KHB. Therefore, strategy of feeding the substrate was attempted to increase the 2-KHB production to avoid substrate inhibition. The 50 mM formaldehyde was supplemented into the reaction solution in batches to sustain a low concentration (no more than 150 mM), and 50 mM pyruvate was also added to avoid the exhaustion every 10 min up to 1 h (final concentration = 400 mM formaldehyde and 500 mM pyruvate). Formaldehyde was accumulated by 9–23.5 mM per batch, and pyruvate was consumed by 11–27 mM, and then it was almost exhausted after 1 h. Time-course reaction by feeding substrate was completed after 50 min and produced approximately 124.8 mM (14.6 g L^−1^) of 2-KHB with volumetric and specific productivities of 14.6 g L^−1^ h^−1^ and 583.4 mg mg-enzyme^−1^ h^−1^ (Fig. 4B), which were 1.63-fold higher than no feeding substrates. The mass imbalance between the consumed substrate and the produced 2-KHB is expected to result from the promiscuous reaction of aldolase. In previous study on MBP-EcYfaU, it produced not only 2-KHB but also 4-hydroxy-3-(hydroxymethyl)-2-oxobutanoate (Hernandez et al. 2017). Furthermore, class II pyruvate aldolase can induce an additional aldol condensation not only 4-hydroxy-3-(hydroxymethyl)-2-oxobutanoate but also 4-hydroxy-4-methyl-2-oxoglutarate (Wang et al. 2010b). We showed the side reaction of MBP-DrADL, which produced 2-KHB as the main product and two unknown peaks (Additional file 1: Fig. S1), which were identified as 4-hydroxy-3-(hydroxymethyl)-2-oxobutanoate and 4-hydroxy-4-methyl-2-oxoglutarate, respectively, by LC–MS analysis (Additional file 1: Fig. S2).

2-KHB production as the main production has been performed by MBP-PaADL in the previous study (Jeong et al. 2023). MBP-PaADL^WT^ and MBP-PaADL^V121A/L241A^ produced 51.6 mM and 73.6 mM with specific productivities of 334.0 and 334.6 mg mg-enzyme^−1^ h^−1^, respectively, which at that point were reported as the highest 2-KHB production so far (Table 2). However, MBP-DrADL in the present study produced 2-KHB concentration that, respectively, was 2.42- and 1.69-fold higher, and showed 1.75- and 1.74-fold higher specific productivities than MBP-PaADL^WT^ and MBP-PaADL^V121A/L241A^. However, its volumetric productivity variant was 0.6-fold and 0.4-fold that of MBP-PaADL^WT^ and MBP-PaADL^V121A/L241A^, respectively, owing to the prolonged reaction time during the batch feeding process. MBP-EcYfaU produced the highest concentration of 2-KHB (Hernandez et al. 2017), which was 9.6-fold higher than MBP-DrADL. However, the volumetric productivity and specific productivity of MBP-DrADL were 2.52- and 100.6-fold higher than MBP-EcYfaU, respectively (Table 2). Overall, considering its potential in generating significant quantities of value-added products through aldol condensation reactions using inexpensive compounds, MBP-DrADL thus holds promise as a viable candidate for 2-KHB production in the industrial sector.

**Table 2:** Quantitative 2-KHB production as the main product from formaldehyde and pyruvate by pyruvate aldolases

Enzyme	Substrate [mM]	Product [mM] (g L^–1^)	Volumetric productivity (g L^–1^ h^–1^)	Specific productivity (mg mg-enzyme^–1^ h^–1^)	References
MBP-EcYfaU	Formaldehyde [1000] Pyruvate [1000]	[1196] (140)	5.83	5.83	Hernandez et al. (2017)
MBP-PaADL^WT^	Formaldehyde [100] Pyruvate [200]	[51.6] (6.03)	24.2	334.0	Jeong et al. (2023)
MBP-PaADL^V121A/L241A^	Formaldehyde [100] Pyruvate [200]	[73.6] (8.60)	34.5	334.6	Jeong et al. (2023)
MBP-DrADL	Formaldehyde [100] Pyruvate [200]	[76.5] (8.94)	8.94	357.6	This study
MBP-DrADL^a^	Formaldehyde [100] Pyruvate [200]	[124.8] (14.6)	14.6	583.4	This study

^a^This reaction was performed by batch-feeding substrates

## Conclusion

The new and thermostable pyruvate aldolase MBP-DrADL was isolated and identified. Gene cloning, expression, and purification enabled us to obtain the purified protein of this enzyme, which has excellent enzymatic properties for use in research or industrial applications. MBP-DrADL exhibited higher aldol condensation activity for formaldehyde and pyruvate than previously reported enzymes and showed thermo-stable activity for 4 days at high temperatures (50, 55, 60, and 65 °C). The biosynthesis of 2-KHB using MBP-DrADL was performed using a continuous substrate feeding strategy to avoid substrate inhibition. Under optimized reaction conditions, 2-KHB was produced at a ratio of with 14.6 g L^−1^, constituting a 1.63-fold improvement over the non-batch-feeding reaction. This is the highest 2-KHB production reported so fat and it will apply to the bioconversion of high-value added compounds from cheap building block compounds such as methane, methanol, and methanol-derived formaldehyde. Moreover, this finding may be potentially useful for the development of synthetic bacteria for C1 gas assimilation and production of value-add chemicals in the future. An environmentally friendly process of 2-KHB production using an efficient pyruvate aldolase should be developed using cascade reactions based on this and other enzymes, to ensure the highest product yield under eco-friendly conditions.

### Supplementary Information

**Additional file 1**: **Table S1.** The sequences of DrADL genes and amino acid. **Figure S1.** HPLC chromatogram profiles of the time-course reaction using MBP-EcYfaU (top) and MBP-DrADL (bottom). **Figure S2.** LC-MS analysis of the byproducts obtained from the enzymatic reaction of MBP-DrADL. The *O*-benzylhydroxylamine derivatization mechanism of 4-hydroxy-3-(hydroxymethyl)-2-oxobutanoate (P1) and 4-hydroxy-4-methyl-2-oxoglutarate (P2), and its exact mass. LC-MS chromatogram profiles of 2-KHB production solution using MBP-DrADL.

## Data Availability

All data generated or analyzed during this study are included in this article.

## Abbreviations

MBP-DrADL: Pyruvate aldolase from *Deinococcus radiodurans* with maltose binding protein
2-KHB: 2-Keto-4-hydroxybutyrate
C1: One-carbon
MBP-EcYfaU: 2-Keto-3-deoxy-l-rhamnonate aldolase from *Escherichia coli* with maltose binding protein
EcKHB: 2-keto-4-hydroxybutyrate aldolase from *Escherichia coli* K-12
BtKHG: 2-Keto-4-hydroxyglutarate aldolase from *Bos taurus*
HsKHG: 2-Keto-4-hydroxyglutarate aldolase from *Homo sapiens*
RnKHG: 2-Keto-4-hydroxyglutarate aldolase from *Rattus norvegicus*
MBP-PaADL: Pyruvate aldolase from *Pseudomonas aeruginosa* with maltose binding protein
MBP: Maltose-binding protein
LB: Luria–Bertani
EPPS: *N*-(2-Hydroxyethyl)piperazine-*Nʹ*-(3-propanesulfonic acid )
SDS-PAGE: Sodium dodecyl sulfate polyacrylamide gel electrophoresis
PIPES: Piperazine-*N*,*N*ʹ-bis(2-ethanesulfonic acid)
CHES: *N*-Cyclohexyl-2-aminoethanesulfonic acid
EDTA: Ethylenediaminetetraacetic acid
HPLC: High performance liquid chromatography
