# Supplementary material for: Biotransformation of 2-keto-4-hydroxybutyrate via aldol condensation using an efficient and thermostable carboligase from Deinococcus radiodurans
Source: Bioresour Bioprocess. 2024 Jan 16;11(1):9. doi: 10.1186/s40643-024-00727-x (PMC10992282; doi:10.1186/s40643-024-00727-x)
Supplement: Supplementary file 1 — Additional file 1: Table S1. The sequences of DrADL genes and amino acid. Figure S1. HPLC chromatogram profiles of the time-course reaction using MBP-EcYfaU (top) and MBP-DrADL (bottom). Figure S2. LC-MS analysis of the byproducts obtained from the enzymatic reaction of MBP-DrADL. The O-benzylhydroxylamine derivatization mechanism of 4-hydroxy-3-(hydroxymethyl)-2-oxobutanoate (P1) and 4-hydroxy-4-methyl-2-oxoglutarate (P2), and its exact mass. LC-MS chromatogram profiles of 2-KHB production solution using MBP-DrADL. [file 40643_2024_727_MOESM1_ESM.docx]

**Additional file**

**Biotransformation of 2-keto-4-hydroxybutyrate via aldol condensation using an efficient and thermostable carboligase from *Deinococcus radiodurans***

Yeon-Ju Jeong^1†^, Min-Ju Seo^2†^, Bong Hyun Sung^3*^, Jeong-Sun Kim^4*^, and Soo-Jin Yeom^1,2*^

**Table S1.** The sequences of DrADL genes and amino acid

| **Enzyme**  **(Accession No.)** | **Nucleotide sequence** | **Amino acid sequence** |
| --- | --- | --- |
| **DrADL**  **(AAF12475.1)** | ATGCCGCAACCGATGAAGCTGGACCCGCTGAGCAACACCTTCAAGCACGCGCTGGCCGGGGGACGGCCCCAGATCGGTTTGTGGCTGGGCTTGGCCGACCCCTACTGCGCCGAAATCTGCGCTGGGGCGGGCTTCGACTGGCTGCTGATCGACGGCGAGCACGCGCCCAACGATGTCCGCAGCACGCTGGCGCAATTGCAGGCGCTGGCAGCTTATCCCGTCGCTCCCGTCGTGCGGCCCCCGGTGGGCGACACGCACCTCATCAAGCAGTACCTCGACCTCGGCGTGCAGACGCTGCTCGTGCCGATGGTGGACACGCCGGAGCAGGCGCGGCAGCTGGTGCAGGCGACCCGCTATCCGCCGCAGGGCATTCGCGGGGTGGGCAGCGCCTTGGCCCGCGCCTCGCGCTGGAACGCGGTGCCCGACTACCTTACGCGGGCGAACGACGAAATCTGCTTGCTGGTGCAGGTCGAGTCGCGCCTGGGGCTGGAAAATTTGGACGAAATTGCAGCGGTAGAAGGCGTAGACGGCGTGTTCATCGGCCCCGCTGACCTCAGCGCCAGCCTGGGGCACCTGGGCCACCCCGGACATCCCGACGTGGCACAGGCCATTGAGGACGCTCTGCGACGCATTGTGGGTGCGGGCAAGGCGGCGGGCATCCTCAGCGCCGACGAGCGGCTGGCGCGGCATTATCTGGCCCTCGGCGCGACCTTCGTGGCGGTGGGGGTGGACACCACGTTGCTCGCCCGCGCAGCCCGGACGCTGGCGGCGAGTTTCAAGGACAAAAGCCGTGAGGAGGCTGAGCCGGAGCCGCAGGGCGGCAGCGTGTATTAA | MPQPMKLDPLSNTFKHALAGGRPQIGLWLGLADPYCAEICAGAGFDWLLIDGEHAPNDVRSTLAQLQALAAYPVAPVVRPPVGDTHLIKQYLDLGVQTLLVPMVDTPEQARQLVQATRYPPQGIRGVGSALARASRWNAVPDYLTRANDEICLLVQVESRLGLENLDEIAAVEGVDGVFIGPADLSASLGHLGHPGHPDVAQAIEDALRRIVGAGKAAGILSADERLARHYLALGATFVAVGVDTTLLARAARTLAASFKDKSREEAEPEPQGGSVY |


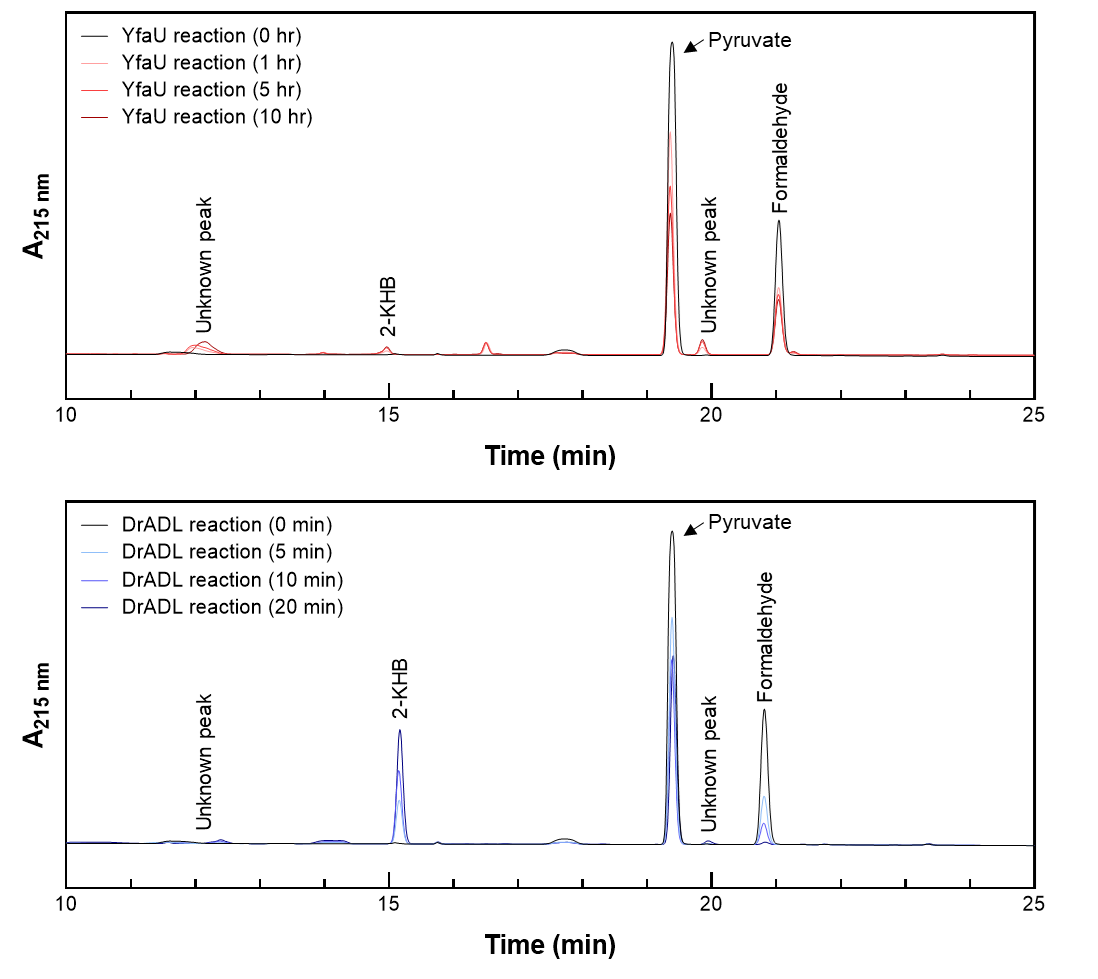


**Figure S1.** HPLC chromatogram profiles of the time-course reaction using MBP-EcYfaU (top) and MBP-DrADL (bottom).


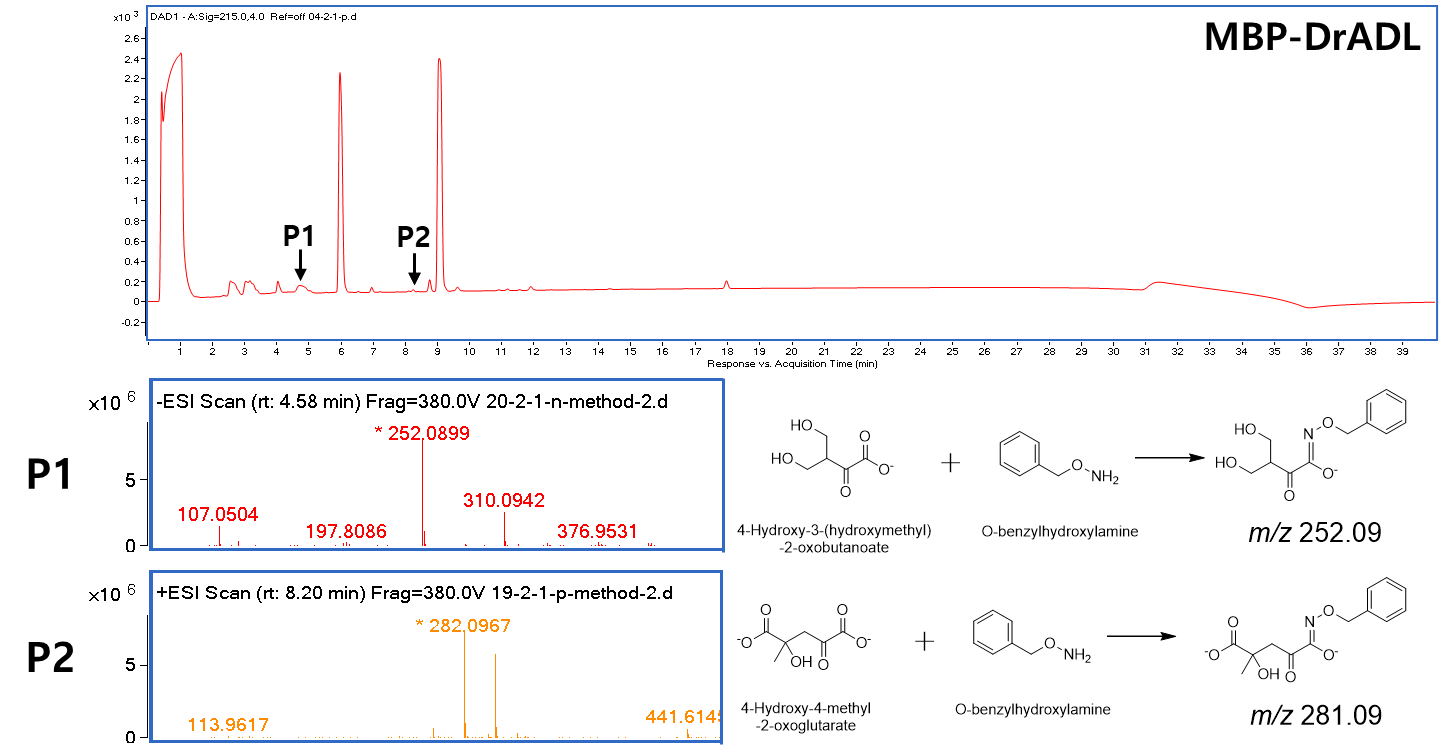


**Figure S2. LC-MS analysis of the byproducts obtained from the enzymatic reaction of MBP-DrADL.** The *O*-benzylhydroxylamine derivatization mechanism of 4-hydroxy-3-(hydroxymethyl)-2-oxobutanoate (P1) and 4-hydroxy-4-methyl-2-oxoglutarate (P2), and its exact mass. LC-MS chromatogram profiles of 2-KHB production solution using MBP-DrADL.
